# Specific cancer rates may differ in patients with hereditary haemorrhagic telangiectasia compared to controls

**DOI:** 10.1186/1750-1172-8-195

**Published:** 2013-12-20

**Authors:** Anna E Hosman, Hannah L Devlin, B Maneesha Silva, Claire L Shovlin

**Affiliations:** 1 NHLI Cardiovascular Sciences, Imperial College London, London, UK; 2 HHTIC London, Respiratory Medicine, Hammersmith Hospital, Imperial College Healthcare NHS Trust, London, UK; 3 Academic Medical Center School of Medicine, Amsterdam, Netherlands; 4 Imperial College London School of Medicine, London, UK; 5 Barts and the London School of Medicine and Dentistry, London, UK

**Keywords:** Breast, Colorectal, Liver, Lung, Prostate, Endoglin, ALK1, SMAD4, Smoking, Metastases

## Abstract

**Background:**

Hereditary haemorrhagic telangiectasia (HHT) is inherited as an autosomal dominant trait, affects ~1 in 5,000, and causes multi-systemic vascular lesions and life-limiting complications. Life expectancy is surprisingly good, particularly for patients over 60ys. We hypothesised that individuals with HHT may be protected against life-limiting cancers.

**Methods:**

To compare specific cancer rates in HHT patients and controls, we developed a questionnaire capturing data on multiple relatives per respondent, powered to detect differences in the four most common solid non skin cancers (breast, colorectal, lung and prostate), each associated with significant mortality. Blinded to cancer responses, reports of HHT-specific features allowed assignment of participants and relatives as HHT-subjects, unknowns, or controls. Logistic and quadratic regressions were used to compare rates of specific cancer types between HHT subjects and controls.

**Results:**

1,307 participants completed the questionnaire including 1,007 HHT-subjects and 142 controls. The rigorous HHT diagnostic algorithm meant that 158 (12%) completed datasets were not assignable either to HHT or control status. For cancers predominantly recognised as primary cancers, the rates in the controls generally matched age-standardised rates for the general population. HHT subjects recruited through the survey had similar demographics to controls, although the HHT group reported a significantly greater smoking habit. Combining data of participants and uniquely-reported relatives resulted in an HHT-arm of 2,161 (58% female), and control-arm of 2,817 (52% female), with median ages of 66ys [IQR 53–77] and 77ys [IQR 65–82] respectively. In both crude and age-adjusted regression, lung cancers were significantly less frequent in the HHT arm than controls (age-adjusted odds ratio 0.48 [0.30, 0.70], p = 0.0012). Breast cancer prevalence was higher in HHT than controls (age-adjusted OR 1.52 [1.07, 2.14], p = 0.018). Overall, prostate and colorectal cancer rates were equivalent, but the pattern of colorectal cancer was modified, with a higher prevalence in younger HHT patients than controls.

**Conclusions:**

These preliminary survey data suggest clinically significant differences in the rates of lung, breast and colorectal cancer in HHT patients compared to controls. For rare diseases in which longitudinal studies take decades to recruit equivalent datasets, this type of methodology provides a good first-step method for data collection.

## Introduction

Hereditary haemorrhagic telangiectasia (HHT, also known as Osler-Weber-Rendu syndrome) is inherited as an autosomal dominant trait, and affects approximately 1 in 5,000 people [1-3]. Affected individuals have multi-systemic vascular lesions that cause major morbidity and mortality [4-6]. Telangiectasia in the nasal mucosa and gastro-intestinal tract frequently haemorrhage leading to chronic iron deficiency anaemia and often transfusion-dependence [1,3,7,8]. Increasing age is associated with increasing severity and prevalence of telangiectasia [3,6], gastrointestinal bleeding [3,8], and comorbidities. Pulmonary, cerebral, spinal and hepatic arteriovenous malformations (AVMs) affect high proportions of patients with HHT, and commonly cause complications including haemorrhagic [9], ischaemic [10] and infective strokes; [10-12] other major haemorrhage; [13] and maternal death in pregnancy [14]. Hepatic AVMs may result in high output cardiac failure, and intractable complicated portal hypertension requiring liver transplantation [15,16]. Additional HHT-related pathologies include pulmonary arterial hypertension (PAH) when the prognosis appears worse than for patients with PAH due to *BMPR2* mutations [17], a higher risk of venous thromboemboli (enhanced in the setting of iron deficiency) [18,19], and for patients with *SMAD4* mutations, colon cancer and other gastrointestinal cancers related to their juvenile polyposis [20,21]. Life-long monitoring and treatment is often needed. Additionally, many patients report not taking secondary prophylaxis such as anti-platelets and anti-coagulants in view of the perceived risk of precipitating haemorrhage [22].

It would be reasonably expected that patients with such severe potential disease complications, apparently increasing with age, should have higher mortality rates than the general population. Life expectancy data demonstrate a higher mortality rate in HHT patients under 60 years of age, consistent with early mortality due to AVMs, especially cerebral AVM bleeds in childhood and young adults, and pregnancy-related deaths [1,23]. In one study, a retrospective analysis of Italian HHT patients’ parents, increased mortality was demonstrated across all age groups [23]. However, in a 30 year prospective study in Denmark there was no evidence for an increase in mortality in HHT patients older than 60 years of age [1]. Although awaiting peer review, more recent data on North American and European cohorts, each of approximately 600 HHT patients or parents, also suggest surprisingly good survival rates [24,25].

Amongst the explanations for the surprising life expectancy data could be that HHT-related mortality is offset by a reduction in deaths from more common diseases. Different rates of heart disease were proposed some years ago, though never formally published [26], and are the subject of a separate manuscript in preparation. Based on personal and family histories from patients attending our specialised HHT service, we hypothesised that HHT patients may have less frequent life-limiting cancers.

Testing such a hypothesis in a rare disease population is not simple. To provide preliminary data in a human population even for the most common cancers such as breast, colorectal, lung and prostate cancer, carries major statistical and logistic difficulties. First, incidence rates (30–50 per 100,000 per annum for lung and colorectal cancers) are prohibitively small for realistic prospective studies in a rare disease population such as HHT. To generate sufficiently sized cohorts for any form of analysis requires pooling of cohorts from different geographical regions. This introduces variance through combining data from genetically unrelated populations, with differing risk factor exposures, and spanning time periods with varying incidence rates [27,28]. As a result, to have sufficient power to detect reductions in cancer rates requires population sizes of many thousands. Additionally, prior fatalities from life-limiting cancers mean that affected individuals may not survive to provide retrospective data at the point of clinic review or questionnaire: in the UK, 5 year survival following breast and prostate cancer is over 80%, but for colorectal cancer, just over 50%, and for lung cancer, less than 10% [28]. Animal models are therefore favoured, but while instructive in specific settings, such models cannot provide an integrated picture of the lifetime exposure risks for people in the setting of the repertoire of human genomic variation.

To design a study to test our hypothesis that cancer incidence may be reduced in HHT, and provide data to allow realistic power calculations to be performed for future studies, we developed an online questionnaire. This extended the techniques we used to capture fatal HHT cerebral haemorrhages [29], and maternal deaths in pregnancy [14], by allowing each individual to provide data on multiple family relatives. This method presents a means of determining cancer rates at lower respondent/proband numbers than if only a single case per respondent was captured; inclusion of relevant questions regarding other family members allows identification of relatives that could have been reported on multiple occasions so allowing each to be captured only once. Questionnaire data are inevitably weakened by the self reported nature, but comparison of subject and control groups ascertained in comparable manners provides an opportunity to compare rates, even if these may not be formally assigned to classical incidence or prevalence rates that demand pre-defined populations.

Here we report a questionnaire-based study, which provides interesting suggestions that specific cancer types may differ between people affected with HHT and controls.

## Methods

### Study design

To capture cancer-histories in an unbiased manner, relevant questions were incorporated into a wider ethically-approved survey. Power calculations (detailed below) indicated that to distinguish incidence rates of the four most common cancer subtypes would require unrealistic response rates, so the study was designed to capture data on multiple relatives per respondent. The basic study design has been reported previously [22,30]. Briefly, in order to prevent participants altering their answers to conform to their guess of what the research hypothesis was, (hypothesis guessing), multiple questionnaires were incorporated into a single survey of questions regarding health and treatments for people with HHT and general population controls. As described elsewhere [22,30], the questionnaire was approved by the NRES Committee East Midlands-Derby 1 Research Ethics Committee, and distributed by post, using the Imperial College London HHTIC London Clinical Service databases (2001 to present), during attendance at the HHT clinics, and advertised by the HHT Foundation International [22,30,31].

Study design allowed participants the option of pausing whilst completing the questionnaire and continuing at a later time point, to optimise data collection and survey completion rates. Generic questions included in the analyses for this study were age, gender, and HHT-related questions which would permit independent assignment of the respondent’s HHT-status based on the Curaçao criteria [32], and allowed HHT-affected respondents to report which parent and grandparent had HHT. Additional questions addressed personal cancer history, family cancer history, and prevalence of carcinogenic risk factors including smoking habits, diet, and industrial exposure to chemicals. These questions were not asked for the relatives due to the excessive number of questions this would have entailed, and the likelihood that no data would have been gathered as participants would have decided to stop the questionnaire. Specific relatives’ questions were therefore limited to age, gender, relationship, if HHT was known to be present, types of cancer present, age at first cancer, and if HHT affected management (if relevant) of cancer treatment (see Additional file 1 for exact wording). Free text options were provided allowing additional details to be reported.

Questions specifying particular cancers targeted the 20 most prevalent cancers in the western world [27,28] with drop down boxes for 5 or 10 year age periods, and each of the specified cancers: skin (basal, squamous, melanoma, other, unsure), and non skin cancers (brain, bladder, breast, cervical, colorectal, kidney, leukemia, liver, lung, lymphoma, malignant melanoma, mesothelioma, mouth, myeloma, oesophagus, ovary, pancreas, prostate, stomach and uterus: see Additional file 1). All questions were standardised, although room was left for personal comment. Study methodology implied that it was not possible to ascertain whether primary or secondary cancers were being reported, but the methodology was identical for control and HHT groupings. In view of reported uncertainty regarding the types of skin cancer present [27,28], it was not the intention to analyse skin cancer data specifically: questions were included however, to ensure these cancers were captured by survey questions before non skin cancers were reported.

### Power calculations

In view of the varied pathogenic mechanisms involved in cancer subgroups, the primary study outcome was specific cancer types, namely the four most common non skin cancers in the UK: breast, colon, lung and prostate. Power calculations were performed assuming each respondent would report raw cancer data on seven unique individuals (grandparents, parents, siblings, and self); an average age of 55; equal gender distributions; and used incidence rate standard deviations of 9.0/100,000 (the maximum for the four cancers listed above) [27]. Such calculations suggested that with 1,000 responses divided between HHT and non-HHT respondents, the study would have 80% power to detect a difference of 0.76/100,000 in incidence rates for lung (or colon) cancer. Since respondents and relatives would include men and women, fewer individuals would be captured for detection of gender-specific cancers. However, the two fold higher rates in the specific sex incidence rates for breast and prostate cancer [27] rendered the calculations for colorectal cancer broadly comparable.

Data for this study were downloaded on 30.6.2012, when 1,307 individuals had responded. Although the survey remained open (for increasingly limited question sets addressing outstanding medical questions) for a further 10 months, only 118 further individuals started the survey in this period.

### Patient population

#### ***Ascertainment of HHT status***

**Figure 1:**
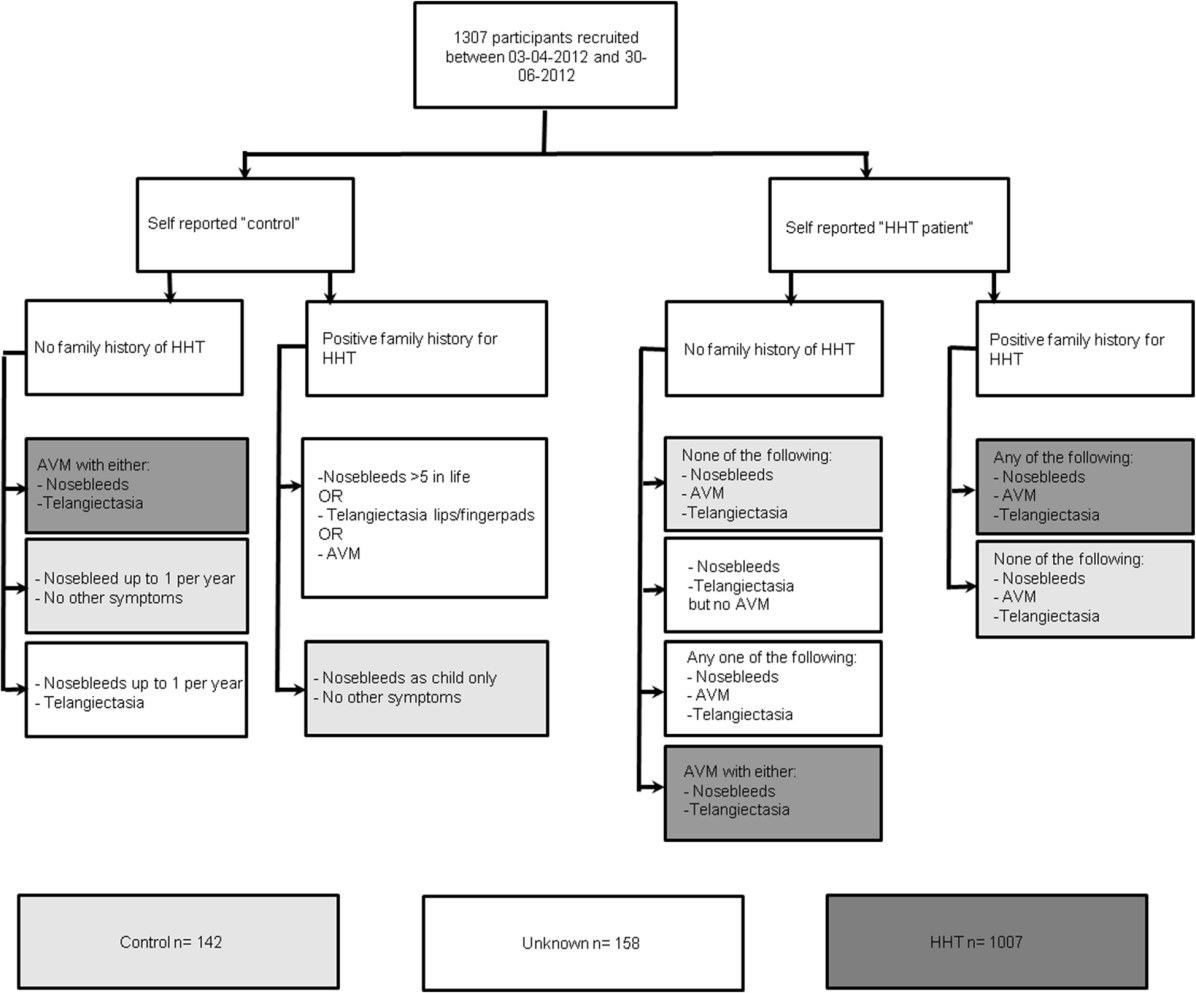
**Stratification of diagnostic assignments.** Flow chart indicating the application of the Curaçao Criteria to Survey Respondents, stratified by whether they reported themselves as having HHT, and by the presence or absence of a family history.

All patients self reported their HHT status, but it was critical to ensure that patients with HHT but unaware of their final diagnosis were not assigned control status, and conversely, that over-exuberant use of the HHT label was not allowed to result in inappropriate assignment of HHT. Therefore, questions were included to address the Curaçao criteria [32] which have been recently validated by a major molecular study [33]. The criteria are nosebleeds, mucocutaneous telangiectasia, visceral involvement (most commonly gastrointestinal telangiectasia, or AVMs at specified sites), and family history. Details of the precise question wording are provided in the Additional file 1. To avoid bias, the telangiectasia question offered a variety of potential sites for “red spots” in tick box options, only two of which were to be considered as HHT telangiectasia (see Additional file 1). A positive family history was defined by a blood relative who had been diagnosed with HHT. Following data download, and prior to analyses of any cancer-related questions, all HHT-diagnostic questions were reviewed independently by two members of the HHT study team, to allow assignment of status as “HHT-subject,” “control”, or “unknown” based on the Curaçao criteria [32], and schematic in Figure 1. The senior author reviewed all assignments. Participants assigned to the “unknown” group were excluded from further data analysis.

In order to capture data on cancers with low incidence and high mortality rates, information on family members provided by the participants was used. Where the respondent had HHT, the questions on which parent and grandparent had HHT allowed deduction of which did not, assuming autosomal dominant inheritance as present in all reported HHT cases to date. Thus for HHT respondents, where it was known which side of the family HHT came from, HHT relatives could then be assigned as “HHT-subject,” “control”, or “unknown”. Specific care was taken to avoid under-diagnosis of HHT that was not yet manifest, thus controls were only selected from the side of the family without HHT. The status of “HHT-patient” or “control” (from HHT unaffected family branches) allocated to family members was stringently assigned prior to analyses of any cancer-related questions. A subgroup of participants reported data during a period when software data collection did not record the age of their parents. For this subgroup, other age data (on themselves, their grandparents, their siblings and other deceased relatives) were complete. For these respondents, parental ages were estimated based on the mean age of mothers at first childbirth using published data for the years 1970, 1980, 1990, 2000, 2003 and 2009 [34]. By cross referencing the names of the oldest patient known in the family to have HHT, and geographical location of the reported relatives, we were able to avoid double-counting relatives reported by multiple respondents. All status assignments were concluded blinded to other demographic and cancer data.

### Statistical methods

Basic demographic variables were calculated using STATA IC versions 11 and 12 (Statacorp, Texas), and Graph Pad Prism 5 (GraphPad Inc, US). An estimate of cancer rates per 100,000 people per year was calculated by adjusting for the specific population gender distribution and median age at the diagnosis of cancer. Given the inherent limitations of survey methodology, to assess if these estimates may be realistic, calculations from cancer data reported for controls in the current survey were compared to the 2008 age-standardised rates *(*ASRs) reported for the Developed World by Globocan [27].

To address whether there may be a difference in rates between the HHT patients and controls captured in comparable methods using the current methodology, two way comparisons between HHT and control groups were performed using Mann Whitney, examining only survey respondents, only relatives, and combined data from all respondents and relatives. Each specified cancer type was used in turn as the dependent variable in logistic regression. Age-adjusted odds ratios for HHT status were calculated by performing logistic regression simultaneously examining the effect of age and HHT status on each specified cancer: p values for contribution from HHT status were calculated post estimation using the non parametric Wald test which makes no assumption about independence of variables. To estimate age-standardised rates for graphical presentations, each individual’s age was assigned to all of the 1–10 decades of life they had achieved, and cancers attributed to the decade in which they occurred. Thus almost all individuals provided more than one decade of life for analyses. Age adjusted rates were calculated for cancers where ages were specifically known, but inclusion of cancers where uncertain ages were spread equally across age groups did not materially alter the relationships.

## Results

### Survey population characteristics

At the time of data download, 1,307 participants had completed the questionnaire. Evaluation of HHT diagnostic criteria, as detailed in Figure 1, resulted in assignment of 1,007 with HHT, 158 unknowns (excluded from further analyses in this study), and 142 controls.

As demonstrated in Table 1, there was no difference in general demographics between HHT and control participants. Median ages were 55ys (range 18–90, interquartile range (IQR) 46-64) and 53ys (range 21–86, IQR 42–61) respectively; 65% of respondents were female (655/1012, [64.7%] HHT; 92/142 [64.8%] control); and there was also no difference in general demographics such as the international region of origin; diet as assessed crudely by vegetarian status/red meat intake; alcohol intake; or exposure to chemicals (Table 1). For smoking, similar percentages were current or former smokers (315/1007 [31%] HHT; 39/142 [27%] controls. Most were cigarette smokers, and most had stopped smoking by the time of the survey (Table 1). However, the smoking habit in terms of pack years smoked per smoker was significantly higher for HHT respondents than controls (Table 1). Crude cancer rates for the two populations are presented in Additional file 2: Table S1.

**Table 1:** Demographics for HHT and control survey respondents

	**Control**				**HHT**				**Total**				**Mann Whitney**
	**Total**	**Count**	**Mean**	**SD**	**Total**	**Count**	**Mean**	**SD**	**Total**	**Count**	**Mean**	**SD**	**p value**
Gender (% female)	142	92	0.65	0.48	1007	654	0.65	0.48	1149	746	0.65	0.48	0.99
North America/Europe	142	124	0.87	0.33	1007	910	0.9	0.29	1149	1034	0.9	0.29	0.25
Australia/New Zealand	142	3	0.02	0.14	1007	42	0.04	0.2	1149	45	0.039	0.19	0.24
Asia	142	1	0.01	0.08	1007	4	0.004	0.06	1149	5	0.004	0.07	0.6
South America	142	0	0	0	1007	4	0.004	0.06	1149	4	0.004	0.06	0.45
Africa	142	0	0	0	1007	2	0.002	0.04	1149	2	0.0017	0.04	0.59
Current or former smoker	142	39	0.27	0.45	1007	315	0.31	0.46	1149	354	0.31	0.46	0.38
Current smoker	142	8	0.06	0.23	1007	67	0.07	0.25	1149	75	0.065	0.25	0.66
Former smoker	142	31	0.22	0.42	1007	248	0.25	0.43	1149	279	0.24	0.43	0.49
Passive smoker	142	1	0.01	0.08	1007	22	0.02	0.15	1149	22	0.019	0.14	0.24
Cigarettes	142	38	0.27	0.44	1007	308	0.31	0.46	1149	346	0.3	0.46	0.37
Number per week	38	38	0.31	0.23	308	308	0.34	0.33	346	346	38.1	21.4	0.4
Years smoked	38	38	15.8	13.5	308	308	17.3	11.5	346	346	17.1	13.4	0.37
Pack years per smoker	38	38	25.4	35.8	308	308	35.4	40	346	346	34	38.7	0.01
Cigars	142	3	0.02	0.14	1007	22	0.022	0.15	1149	25	0.022	0.15	0.96
Pipes	142	3	0.02	0.14	1007	18	0.018	0.13	1149	21	0.019	0.13	0.99
Other	142	1	0.01	0.84	1007	14	0.014	0.12	1149	15	0.013	0.11	0.5
Non vegetarian	130	123	0.95	0.23	969	929	0.96	0.2	1094	1047	0.96	0.21	0.51
Red meat 3x per week	130	75	0.58	0.5	950	542	0.57	0.5	1075	614	0.57	0.5	0.89
Industrial exposures	130	121	0.93	0.25	964	885	0.92	0.27	1090	1002	0.92	0.27	0.62
Alcohol	130	53	0.41	0.49	969	342	0.35	0.48	1099	395	0.36	0.48	0.22
Alcohol units per day	130	84	0.65	0.95	970	518	0.53	0.87	1095	602	0.55	0.88	0.17

Total: number of respondents reporting presences, absence or distribution of variable. Count: number of respondents with specified variable- demographics; originating in stated region; or describing stated intakes/use/exposures. SD, standard deviation.

### Relatives and combined groupings

The survey also captured cancer data on 4,930 grandparents and parents. 1,154 were reported as HHT-affected. 2,675 relatives could be confidently assigned as controls as they were either relatives of control respondents, or from non-HHT branches of HHT families. The remaining relatives (n = 1,101) could not be assigned as they were in potentially HHT-affected branches of the families, and the diagnosis of HHT may not yet have manifest [6-10], or they had been potentially reported by other survey respondents. Data from these relatives were therefore not analysed.

The respective median ages of survey respondents were 53ys [IQR 42–61] for controls and 55ys [IQR 46–64] for HHT subjects. Ages of reported relatives were higher at median 77ys [IQR 67–82] for controls; median 72ys [IQR 62–82] for HHT-affected relatives. Combining data of participants and relatives resulted in a control-arm of 2,817 (52% female, median age 77ys [IQR 65–82]), and HHT-arm of 2,166, (58% female, median age 66ys [IQR 53–77]).

### Validation of survey methodology using control data

To validate the study methodology, the estimated cancer rate (per 100,000 patients per year) was calculated for the control group, and compared to ASRs for the Developed World from Globocan [27], recognising that Globocan ASRs were for primary cancers at the designated sites, whereas study methodology would include reports of metastatic cancers. For the 18 most common non-skin cancers, Table 2 presents the crude data; adjustments for a population of average age 77ys, 52% female; and the ratios of the observed ASR/expected ASR. These ratios ranged from 0.43 to 2.3 (median 1.23). For the 15 “predominantly primary” cancers, the average ratio approximated to 1.0, compatible with robust study methodology. We concluded that while the data in the survey were not from a geographical or numerically-defined population, and while there were inevitably concerns about self reported data, nevertheless, the survey data for controls were reflective of the cancer rates in the general population.

**Table 2:** Calculation and comparison of age standardised rates (ASRs) for cancers in the control arm, compared to reported general population data

	**Control survey population**			**Globocan ASR ^**			**Ratio**
	**Cases**	**Cases per 100,000**	**ASR per 100,000^**	**ASR (men)**	**ASR (women)**	**ASR if 52% female**	**Survey ASR/Globocan ASR**
Bladder	9	319	4.1	16.3	3.6	9.7	0.43
Brain	21	745	9.7	5.8	4.4	5.1	1.91
Breast	91	3230	42.0	0.0	66.4	34.5	1.22
Cervical	14	497	6.5	0.0	9.1	4.7	1.36
Colorectal	56	1988	25.8	37.7	24.3	30.7	0.84
Kidney	11	390	5.1	11.9	5.9	8.8	0.58
Leukemia	17	603	7.8	9.1	5.9	7.4	1.05
Liver	27	958	12.4	8.2	2.7	5.3	2.33
Lung	106	3763	48.9	47.1	18.8	32.4	1.51
Lymphoma	19	674	8.8	12.5	9.0	10.7	0.82
Mouth	5	177	2.3	6.8	2.3	4.5	0.52
Myeloma	8	284	3.7	3.3	2.2	2.7	1.35
Oesophagus	9	319	4.1	6.5	1.3	3.8	1.09
Ovary	13	461	6.0	0.0	9.3	4.8	1.24
Pancreas	21	745	9.7	8.3	5.5	6.8	1.41
Prostate	56	1988	25.8	61.7	0.0	29.6	0.87
Stomach	39	1384	18.0	16.7	7.3	11.8	1.52
Uterus	20	710	9.2	0.0	13.0	6.8	1.36

General population ASRs were Globocan ASRs for “More Developed Regions”, 2008 [27]. The study data represent cancer cases in 2817 control participants or relatives, with an average age of 77 ys, and 52% female. ^ Calculated assuming median age of 77 ys.

**Figure 2:**
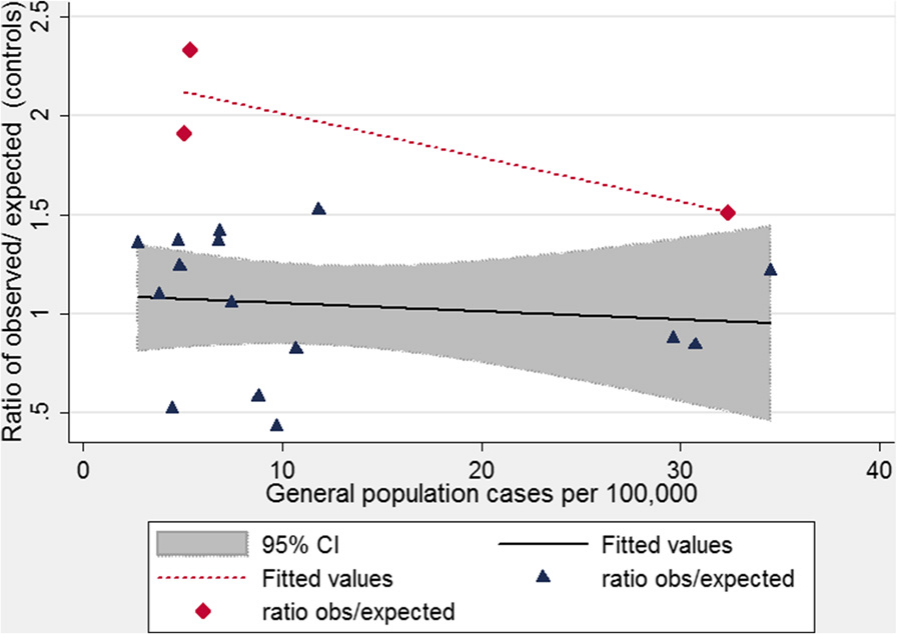
**Validation data of cancer rates in controls.** Comparison of age-standardized rates (ASRs) for survey control arm and Globocan ASRs for “More Developed Regions”, 2008. The study data represent cancer cases in 2,817 control participants or relatives, with an average age of 77 ys, and 52% female, and plots the observed/expected ratios presented in Table 2, against the overall frequency of the specified cancer, since variance would be expected to be greater for less common cancers. Data are stratified by whether the cancers are predominantly primary only (navy symbols, black solid line with 95% confidence intervals); or primary and secondary sites (red symbols and red dotted line).

Of the 18 cancer types, three were at common sites of metastatic spread, namely lung, liver and brain. The ratio of ASRs for these cancer types (range 1.5-2.3, median 1.9) was significantly higher than for the other 15 cancer types (range 0.43-1.52, median 1.09, p = 0.013). Figure 2 illustrates the ASR ratios for the two subgroupings, plotted against the frequency of the particular cancer type. Since for the three “primary plus metastatic” sites, the cancers were reported more commonly than expected by primary ASRs, we concluded that the data were compatible with respondents reporting both primary and metastatic cancers for lung, liver and brain.

### Comparison of cancer rates in HHT patients and controls

**Figure 3:**
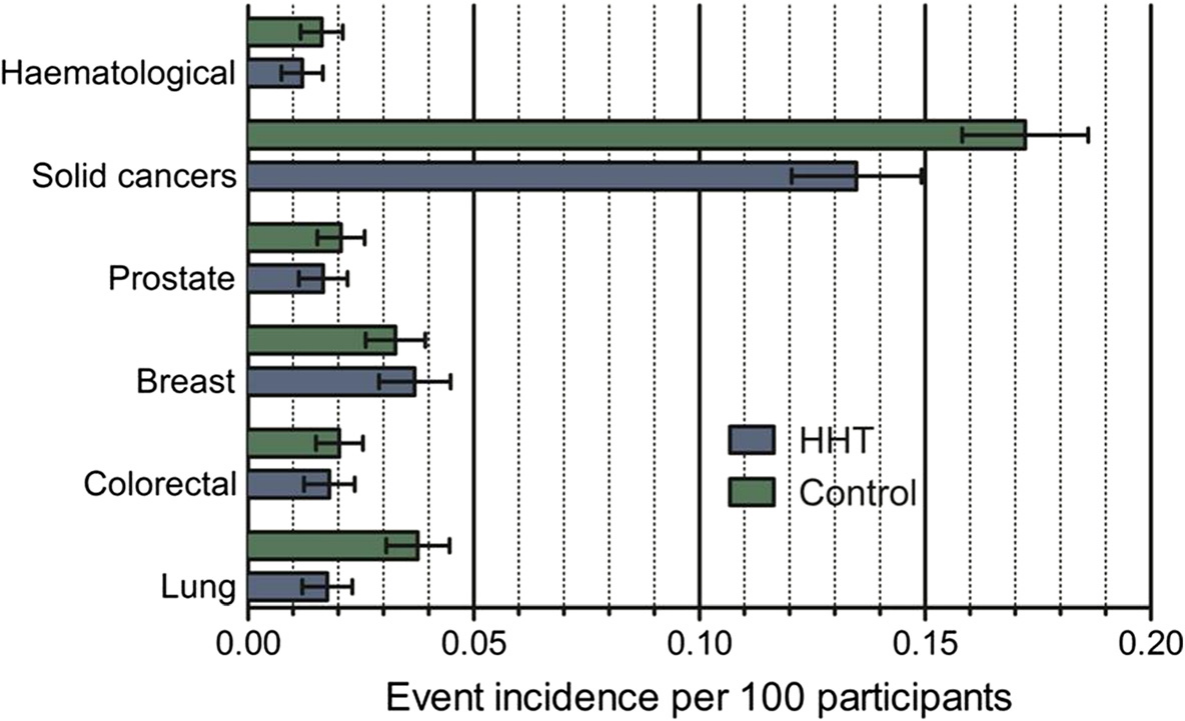
**Reported numbers for haematological and solid cancers.** Data are illustrated for the most common four cancers and haematological cancers, in 2,166 HHT patients and 2,817 controls (Error bars indicate 95% confidence intervals).

Calculated cancer rates were then compared between the survey HHT and control groups. In crude analyses, fewer cancers were reported for HHT (398/2161, 18.4%) than controls (668/2817, 23.7%, p = 0.0012). As noted in Figure 3 and Table 3, in these crude figures, there appeared to be a lower frequency of solid tumours, and specifically of lung cancers in the HHT arm compared to controls. Since primary and secondary lung and liver cancers carry high mortality [28], and the HHT population comprised a greater proportion of respondents (introducing a bias as they needed to survive to the point of survey completion), cancer rates were also examined in the “relatives only” subgroup, representing 1,154 HHT-affected relatives and 2,675 control relatives. This revealed higher rates of these life-limiting cancers than in the younger survey respondents, but again, the crude rates of lung and liver cancer were lower in the HHT group than in controls: Crude liver cancer rates for the relatives-only groups (27 cases in controls, 10 in HHT) were 1,009 and 866 per 100,000 respectively. Crude lung cancer rates for the relatives-only groups (101 cases in controls, 33 in HHT) were 3,775 and 2,860 per 100,000 respectively.

**Table 3:** Crude incidence of the 20 most common non skin cancers in both study arms

	**Controls (n = 2817) †**			**HHT (n =2161) ‡**			
	**Cancer cases**	**Cases per 100,000**	**SEM**	**Cancer cases**	**Cases per 100,000**	**SEM**	**p value**
Bladder	9	320	106	12	5090	153	0.3
Brain	21	746	162	11	463	146	0.21
Breast	91	3230	333	80	3702	406	0.38
Cervical	14	497	133	11	463	146	0.86
Colorectal	56	1988	263	37	1712	279	0.47
Kidney	11	391	118	11	463	146	0.7
Leukemia	17	604	146	7	324	122	0.16
Liver	27	959	184	11	463	146	0.042
Lung	106	3763	359	38	1758	283	<0.0001
Lymphoma	19	675	154	16	740	185	0.79
Melanoma	67	2378	287	45	2082	307	0.48
Mesothelioma	1	36	36	0	0	0	0.38
Mouth	5	178	79	7	324	122	0.3
Myeloma	8	284	100	4	185	93	0.48
Oesophagus	9	320	106	4	185	93	0.35
Ovary	13	462	128	13	602	166	0.5
Pancreas	21	746	162	9	417	139	0.14
Prostate	56	1988	263	33	1527	264	0.22
Stomach	39	1384	220	14	648	173	0.012
Uterus	20	710	158	10	463	146	0.26
All cancers°	668	23713	800	398	22351	834	0.0012

† The control arm represented 142 survey respondents and 2,675 reported relatives. ‡ The HHT arm represented 1,007 survey respondents and 1,154 reported relatives. Note that for stomach and pancreatic cancer, the higher than expected control values (Table 2) suggest that these primary cancers may not have been appropriately assigned by respondents and relatives. ° All cancers includes less common cancers. P value calculated by Mann Whitney.

### Age-adjusted cancer rates

The individuals provided 36,887 separate decades of life for analyses: 15,053 in the HHT arm and 21,834 decades in the control arm. As expected, cancer rates were strongly age-related (p < 0.0001, all cancers). Age adjusted incidence rates were calculated for all cancers combined, and for the most common cancers. These data indicated that after age-adjustment, there was no significant difference in the overall rates of all cancers between HHT and controls (Table 4), but this masked different patterns amongst the four most common cancers: Following age-adjustment, there was no difference in prostate or colorectal cancer rates, but breast cancer was reported more frequently for HHT patients (age-adjusted OR 1.52 (1.07, 2.14), p = 0.018), and lung cancer significantly less frequently for HHT patients (age-adjusted OR 0.48 [0.30, 0.77], p = 0.0023).

**Table 4:** Crude and age adjusted HHT odds ratios for the four most common cancers

	**Crude odds ratio (95% CI)**	**p value**	**Age adjusted odds ratio (95% CI)**	**p value**
All cancers	0.83 (0.72, 0.96)	0.012	1.04 ( 0.90, 1.21)	0.53
Prostate	1.11 (0.71, 1.76)	0.64	1.37 (0.87, 2.19)	0.18
Colorectal	1.04 (0.65, 1.65)	0.89	1.30 (0.81, 2.08)	0.28
Breast	1.18 (0.84, 1.65)	0.35	1.52 (1.07, 2.14)	0.018
Lung	0.38 (0.24, 0.60)	<0.0001	0.48 (0.30, 0.77)	0.0023

Logistic regression was performed in all HHT patients and controls for all cancers, colorectal and lung cancers, in males only for prostate cancer, and in females only for breast cancer. The age adjusted p values for contribution of HHT status were calculated post estimation using Wald test.

The study had not been powered to detect differences in rates of liver cancer, but pooling with reported stomach cancer was considered logical, given stomach cancer was the most generic term available for abdominal cancer in these family reports, and was over-represented in the control group compared to Globocan [28]. Pooled data suggested HHT patients had fewer liver and stomach-designated abdominal cancers than controls (age-adjusted odd ratio 0.51 (0.25, 1.02), p = 0.059) (Table 5).

**Table 5:** Crude and age adjusted HHT odds ratios for specified abdominal cancers

	**Crude odds ratio (95% CI)**	**p value**	**Age adjusted odds ratio (95% CI)**	**p value**
Liver	0.46 (0.18, 1.14)	0.095	0.59 (0.23, 1.49)	0.26
Stomach	0.43 (0.20, 0.95)	0.036	0.53 (0.24, 1.17)	0.12
Liver or stomach	0.51 (0.25, 1.03)	0.059	0.51 (0.25, 1.02)	0.059

Logistic regression was performed in all HHT patients and controls. The age adjusted p values for contribution of HHT status were calculated post estimation using Wald test.

### Patterns of age-related changes

**Figure 4:**
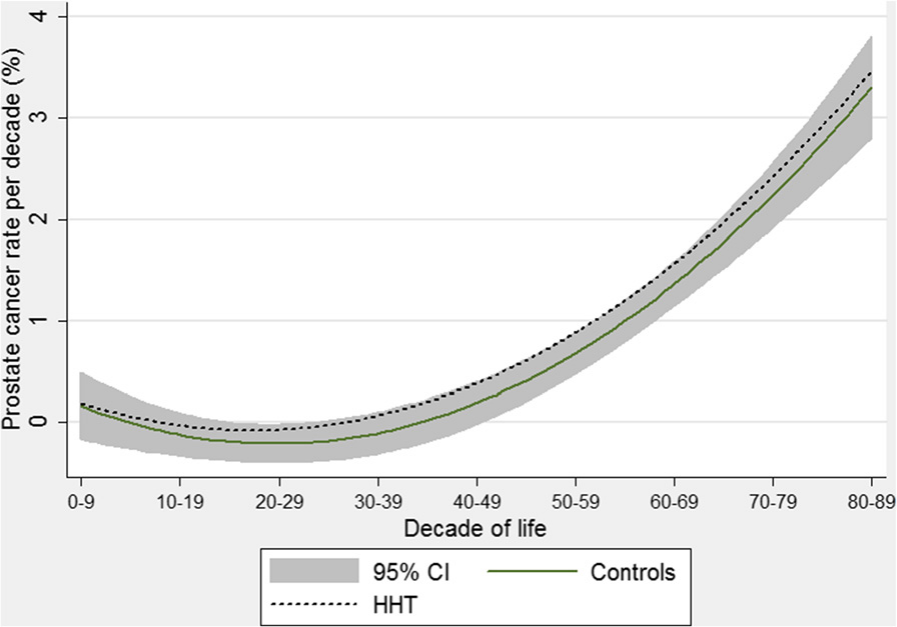
**Age-specific prostate cancer rates.** Quadratic regression plots for male-only HHT patients and controls. Shaded areas indicate 95% confidence intervals.

To examine whether there were trends for differences between the HHT and control groups at specific periods of their lives, quadratic regression was used to present age-related changes graphically. As shown in Figure 4, for prostate cancer, there was an exponential rise in cancer with age in both controls and HHT patients. The best-fit quadratic regression line for HHT patients fitted within the 95% confidence intervals for the best-fit line in the control population. These graphs represent the pattern that would be expected if there were no differences in prostate cancer rates in any age group, between HHT patients and controls.

**Figure 5:**
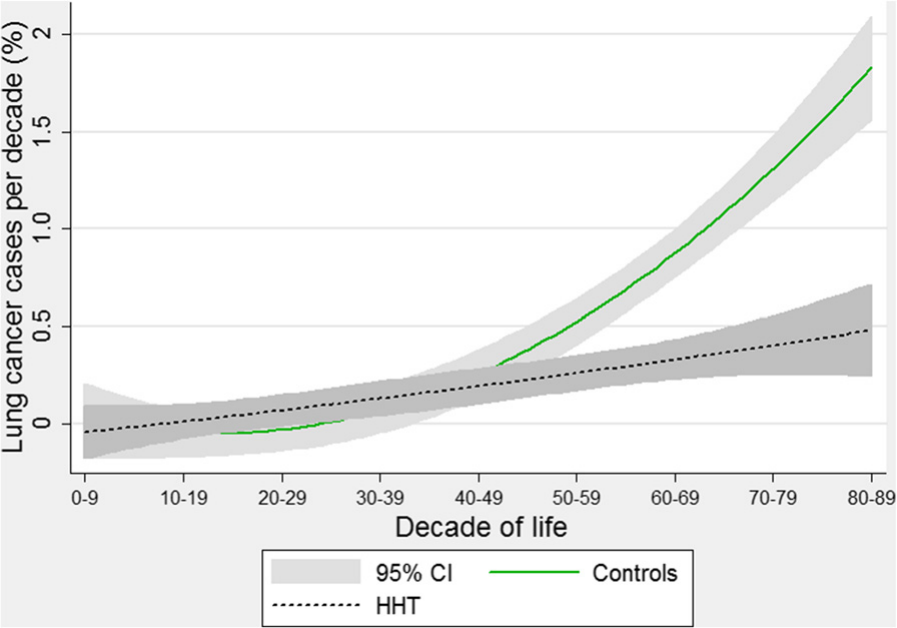
**Age-specific lung cancer rates.** Quadratic regression plots for all HHT patients and controls. Shaded areas indicate 95% confidence intervals. Note primary and secondary lung cancers are not distinguished.

For lung cancer, the control arm again demonstrated an exponential rise with age (Figure 5). In contrast, the best-fit line for lung cancer events in the HHT-arm was more linear, and less steep than the comparable curve for the controls. The 95% confidence intervals for the best-fit curves diverged after the 5th decade of life. These graphs represent the pattern that would be expected if lung cancer (primary or secondary) was less common in older HHT patients compared to equivalently aged members of the general population.

**Figure 6:**
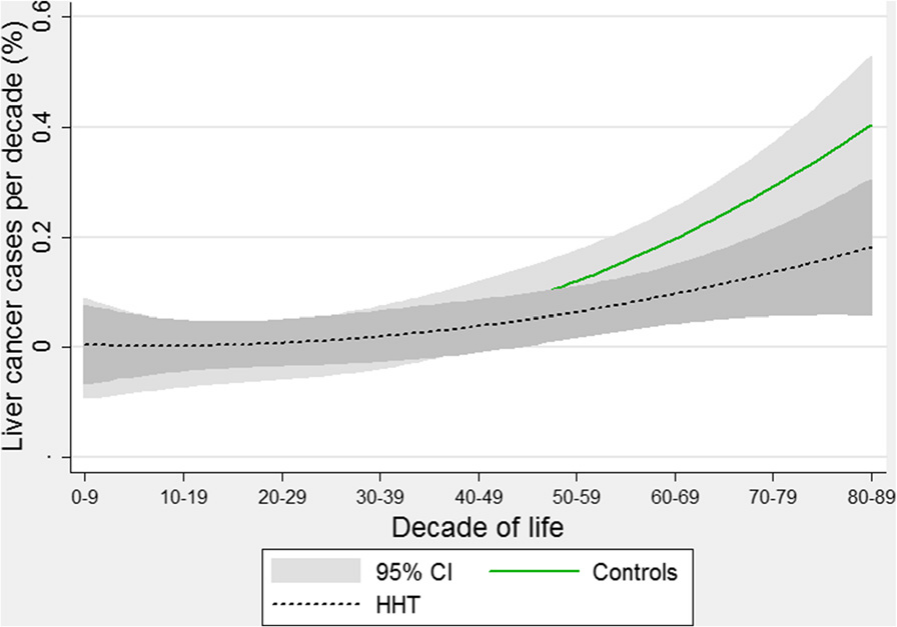
**Age-specific liver cancer rates.** Quadratic regression plots for all HHT patients and controls. Shaded areas indicate 95% confidence intervals. Note primary and secondary liver cancers are not distinguished.

A similar trend was observed for liver cancer (Figure 6), although the study had not been powered to detect a difference in this less common cancer type. With the wider confidence limits, the 95% confidence intervals for the best-fit curves did not quite diverge. Again, these graphs represent the pattern that would be expected if liver cancer (primary or secondary) was less common in older HHT patients compared to equivalently aged members of the general population.

**Figure 7:**
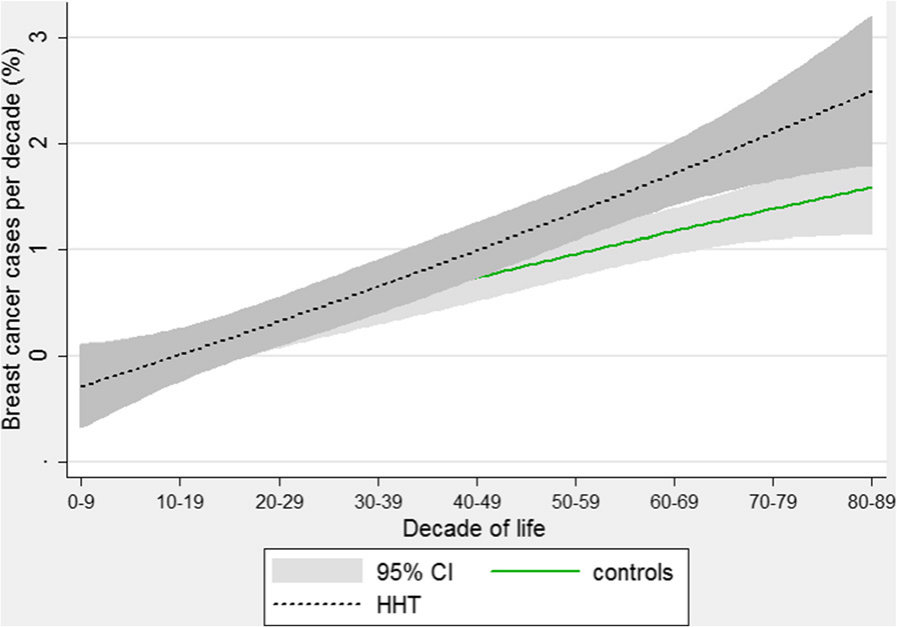
**Age-specific breast cancer rates.** Quadratic regression plots for female-only HHT patients and controls. Shaded areas indicate 95% confidence intervals.

For breast cancer, a different pattern was observed. For both controls and HHT patients there was a more linear increase in breast cancer cases with age (Figure 7). The curves diverged after 50 years of age but in this case, it was the HHT population who showed a greater increase of cancers with age. These graphs represent the pattern that would be expected if breast cancer was more common in older HHT patients compared to equivalently aged members of the general population.

**Figure 8:**
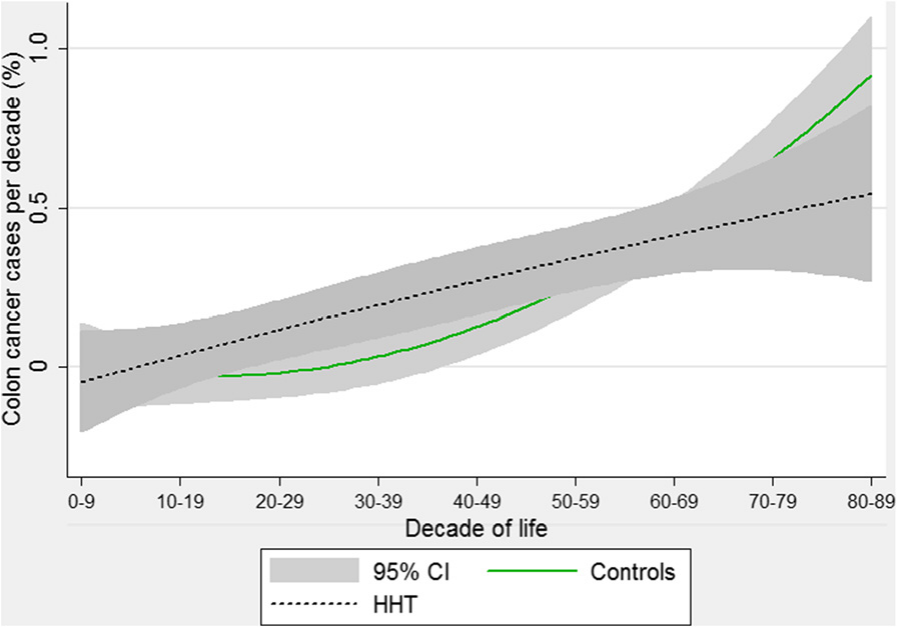
**Age-specific colorectal cancer rates.** Quadratic regression plots for all HHT patients and controls. Shaded areas indicate 95% confidence intervals.

It had been expected that rates of colorectal cancer would be higher in HHT because of the population subgroup with *SMAD4* mutations and juvenile polyposis. Crude and age-adjusted analyses had not revealed an overall difference in colorectal cancer rates between the control and HHT groups, but quadratic regression suggested a bimodal pattern (Figure 8). At younger ages, colorectal cancers were more common in HHT patients, but the rate of rise with age was less steep than for controls, and at older ages, the trend was for fewer cancers in HHT patients.

## Discussion

In this study, using a new tool to capture rates of uncommon conditions within a rare disease population, we demonstrated apparent differences in incidence of particular subtypes of cancer in HHT patients compared to controls. Lung and liver/abdominal cancers appeared to be less prevalent, and breast cancer more prevalent in HHT patients. Overall, given the poorer survival from lung and liver cancer compared to breast cancer, the data could account for the surprisingly good life expectancy in older HHT patients.

The strengths of this study included the use of new methodology, designed as a family-based questionnaire powered to detect differences in rates of the four most common non-skin cancers between HHT patients and controls. The specific questionnaire was strengthened by the design, accessibility of the questions, standardised and objective inclusion criteria applied after data capture, and acquisition of data from a large number of subjects for a rare disease population. Design of the survey prevented “hypothesis guessing” by participants by using questions on other common health issues that concealed the purpose of each section of the survey. Due to the familial nature of the condition, participants exhibited willingness to report detailed data on themselves and relatives, despite being unclear exactly why the questions were being asked. The large control group permitted validation of methodologies by comparing ASRs for specific cancers in the captured controls, to those reported for equivalent geographical populations.

Clearly there are limitations with this type of approach which relies on retrospective recollections with potential bias and honesty of data reporting. In addition, it may be limited by uncertainty on precise details of the HHT diagnosis. This was addressed by not merely using self reported status, but also using a rigorous algorithm that meant that 12% of completed datasets were not assignable either to HHT or control status. While we cannot exclude that some individuals reporting they had AVMs at particular sites, or particular AVM treatments, were wrong, these were never used in isolation for the diagnosis of HHT (Figure 1). Absence of a molecular diagnosis in the majority of cases may be considered a limitation by scientists, but as clinicians recognise, only a proportion of HHT families can receive a molecular diagnostic confirmation. Conversely, given the currently debate regarding the disease-causing status of many missense HHT mutations [35,36], incomplete descriptions of a change in one of the HHT genes were considerably more likely to be misreported than a clinical phenotype that was familiar to the patient. The study was conducted on a predominantly western, English-speaking population aged between 18 and 90 years of age, though cross references were made to general population cancer data from equivalent countries. Detailed smoking and epidemiological habits of relatives were not available, although with the exception of smoking, the control and HHT respondent groups were similar in virtually all demographics analysed. We were particularly concerned with the potential bias of survival to study participation, because lung and liver cancers carry high mortality [28]. Had the reduced number of lung and liver cancers observed in HHT purely been due to survival bias (as participants needed to survive to the point of survey completion), more lung/liver cases should have been found in the HHT “relatives only” subgroup. Since lower rates of lung and liver cancer were reported for HHT relatives than control relatives, we concluded that even allowing for potential survival bias, the data suggested a genuine reduction in these cancers in HHT patients.

From laboratory and animal studies, there are opposing datasets suggesting HHT patients may be at higher or lower risk of cancer and metastases, reflecting the complexity of multistep cancer pathogenesis, and the importance of attempting to obtain data from patients, despite the methodological limitations compared to laboratory analyses. The majority of HHT patients have endoglin or ALK1 mutations, and are haploinsufficient [35], expressing approximately half normal endoglin or ALK1 in activated monocytes, human umbilical vein endothelial cells, and blood outgrowth endothelial cells [35-43]. Over-expression of both endoglin and ALK-1 is seen during tumour development and endothelial cell proliferation where new vessels are formed to support tumour growth [44-53]. Consequences of acute changes in endoglin and ALK1 expression are yet to be fully determined but include modulation of oncogenic genes such H-Ras; [45] DNA repair enzymes [46], apoptosis [47], and resistance to chemotherapy [46]. For metastases, while there are data that acute use of anti-endoglin [47] or anti-ALK1 antibodies [48] attenuate endothelial sprouting and other early angiogenic processes, recent data suggest that long term deficiency may render endoglin deficient mice at enhanced risk of tumour metastatic spread [49], and that endoglin overexpression may be protective [50]. Conversely, there are data that cancer growth is reduced in endoglin ^+/-^ mice [51,52]. Importantly, both endoglin and ALK1 are emerging as successful targets for cancer therapies in the general population: The use of a soluble chimeric protein (ALK1-Fc), an inhibitor of ALK-1, has been shown to result in significant tumour-suppression both in vitro and in vivo [53]. Furthermore, Phase 1 and Phase 2 human trials have been performed with anti-endoglin antibodies with encouraging results [54,55].

Our hypothesis based on clinical observations and the surprisingly good life expectancy data, was that cancer rates would be lower in HHT patients: This interpretation would be in-keeping with the data from the human trials [53-55]. The current study was powered to detect differences in lung cancer rates, and these emerged as significantly lower in HHT patients than controls ascertained using the same methodology. We cannot rule out a chance over-reporting of lung cancers only for the control arm, or that HHT patients who would have gone on to develop either primary lung cancer or lung metastases had already died from HHT or other causes, although in the latter case, as for lung cancer specific mortality above, we would have expected to see a higher rate in the relatives arm, but did not. The risk of primary lung cancer is strongly smoking associated, but it is difficult to attribute the lower rates of lung cancer to reduced smoking, as the data suggest smoking rates were if anything, higher in HHT patients compared to controls. Data from our ongoing 2013 HHT Survey [56] provide a plausible reason: of the first 137 smokers, two (1.5%) stated smoking seemed to start a nosebleed, but 13 (9.5%) stated smoking seemed to stop a nosebleed (Mann Whitney p = 0.0062). We emphasise that the hazards of smoking mean smoking should not be viewed as a “therapeutic” option for HHT nosebleeds- smoking cessation is strongly recommended for HHT patients, as for the general population.

While the current study was underpowered to address liver and other abdominal cancer rates, these too appeared to be reduced. We therefore think it may be relevant that comparisons to age-standardised rates in the general population suggest a significant proportion of reported lung, liver and brain cancers were likely to be metastases from primary cancers elsewhere (Table 2, Figure 2). For lung cancer, we suggest it is possible that overall, HHT patients have natural protection against tumour development in terms of tumour initiation, growth, and/or metastases. Irrespective of the mechanism (s), given the dismal survival rates once lung cancer is present [28], reduced rates of lung cancer could account for the life expectancy paradox evident in the HHT population.

In view of case reports and evidence that colorectal cancer risks are higher for patients with *SMAD4* mutations, we were surprised that the risk of colorectal cancer did not emerge more strongly for participants and/or relatives with HHT. The age-related changes would support an interpretation allowing for an enhanced risk in early life (most likely consequent on *SMAD4* and polyposis predispositions), but possible protection from other forms of colorectal cancer later in life.

Breast cancer was also expected to be higher in HHT patients: As for any discipline in which screening and treatment modalities include exposure to ionising radiation, there are discussions about the degree to which health benefits may be offset by an increase in cancer rates [57-61]. In HHT, this is particularly true for brain, lung and breast tissues which lie within the radiation exposure fields for CT scans and angiographic studies that are essential to treat HHT cerebral and pulmonary AVMs respectively. Furthermore, endoglin, the protein mutated in HHT type 1, has been shown to suppress invasion and metastasis of breast cancer, with lower endoglin expression in the tumour compartment correlating with poorer clinical outcome [54]. Since HHT patients with endoglin mutations express approximately half normal endoglin [37-43], there would therefore be even more reason to predict that breast cancer rates should be higher in HHT patients. However, only a modest increase was observed (age-adjusted OR 1.52 (1.07, 2.14), p = 0.018). Whether this increase would be lessened by reduced radiation exposure is testable, but it is important to recognise that the lifetime risks of breast cancer (<1%) are substantially lower than the risks of strokes, brain abscess, and other complications, which are prevented by PAVM embolisation.

**Figure 9:**
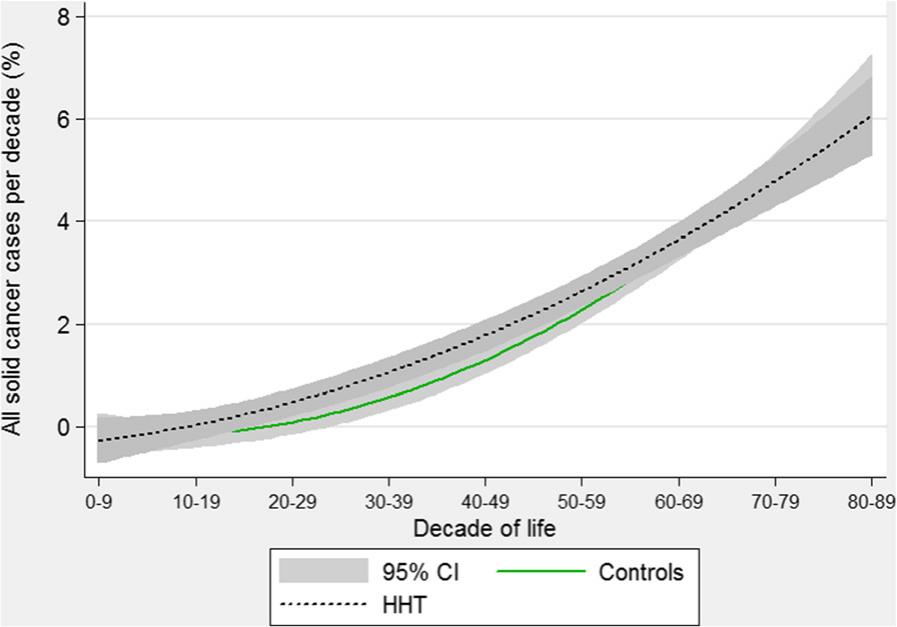
**Age-specific rates of all solid, non-skin cancers.** Quadratic regression plots for all HHT patients and controls. Data include brain, bladder, breast, cervical, colorectal, kidney, liver, lung, pancreas, prostate, stomach uterus, mouth and oesophageal cancers. Shaded areas indicate 95% confidence intervals.

**Figure 10:**
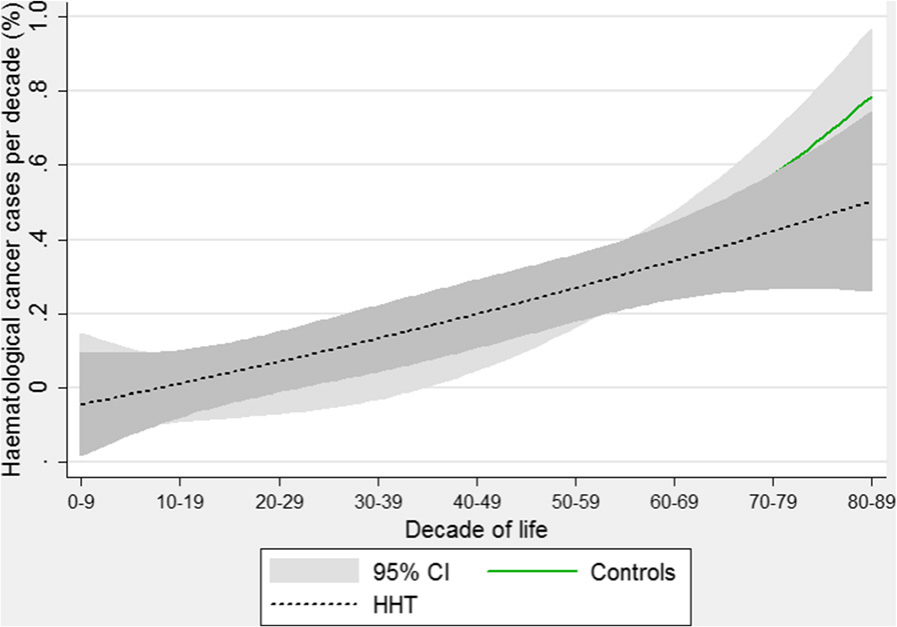
**Age-specific rates of haematological cancers.** Quadratic regression plots for all HHT patients and controls. Data include leukaemia, lymphomas and myeloma. Shaded areas indicate 95% confidence intervals.

Due to the divergent patterns particularly for lung and breast cancer, there were no evident trends comparing all solid cancers (Figure 9). This provides a cautionary note regarding pooling different disease states when faced with the demanding logistical or statistical requirements for studying comorbidities in patients with rare diseases. This could have been done in this study, for example powering the study to detect a difference in “all cancers”, “all solid cancers”, or “all haematological cancers”. There were also no differences in the rates of pooled haematological cancers between HHT patients and controls (Figure 10). Instead of speculating on potential reasons, we prefer to emphasise that the study was underpowered to detect differences even when pooled, and that, as for solid cancers, pooling may have masked important differences between individual cancer types.

### Concluding remarks

Overall, for rare diseases in which longitudinal studies would take decades to recruit equivalent datasets prospectively, we suggest that this type of methodology is a good first-step method for data collection. Rapid high profile advertising in the specific populations (particularly via well established patient support groups with links through email, facebook and twitter to hundreds or thousands of affected/interested potential participants) renders prolonged data capture periods unnecessary. Such a tool provides the opportunity to address comorbidity risk reductions in rare disease populations, instead of risk increases which are easier to address statistically. Providing patients with rapid feedback from their participation in a somewhat arduous questionnaire is likely to increase their willingness to participate in further studies. This is important for rare disease populations where future research studies are likely to target the same patient groups. Additionally, if multiple research questions are addressed in the same survey, this reduces reporter bias, offers opportunities for almost immediate delivery of results that matter to patients [7,22,30], yet could potentially be used to capture data of more interest to researchers than the participants themselves.

For the HHT community, these study results are reassuring on multiple levels, and particularly in terms of absolute lung, breast, brain and colorectal cancer rates given the inevitable speculation regarding potential risks based on available laboratory evidence. We suggest that the findings are also important to the scientific community, as they suggest that HHT patients may be protected from common cancers. Further studies are recommended to assess if factors that may be protecting the HHT population could also be harnessed for the benefit of the general population.

## Abbreviations

ALK1: Activin receptor like kinase 1; ASR: Incidence of cancers per 100,000 people per year; AVMs: Arteriovenous malformations; HHT: Hereditary haemorrhagic telangiectasia; IQR: Interquartile range.

## Competing interests

The authors have no competing interests to declare.

## Author contributions

AEH assisted in design of survey cancer questions, participated in survey testing, recruitment and phenotypic assignments, performed the primary analyses, and drafted the manuscript. HLD participated in survey testing and recruitment, and performed phenotypic assignments. BMS participated in survey testing and recruitment. CLS conceived the study, performed the power calculations, generated the overall study questions and SurveyMonkey questionnaires, participated in phenotypic assignments, performed all population comparison and regression analyses, and wrote the final manuscript. All authors read and approved the final manuscript.

## Supplementary Material

Additional file 1 Extracts from Survey. Click here for file

Additional file 2 Crude incidence of the 20 most common non skin cancers in survey respondents. Click here for file
