# Supplementary material for: Specific cancer rates may differ in patients with hereditary haemorrhagic telangiectasia compared to controls
Source: Orphanet J Rare Dis. 2013 Dec 20;8:195. doi: 10.1186/1750-1172-8-195 (PMC3891994; doi:10.1186/1750-1172-8-195)
Supplement: Additional file 1 — Extracts from Survey. [file 1750-1172-8-195-S1.pdf]

Cancer in hereditary hemorrhagic telangiectasia and controls:
Results from an international survey capturing 4,983 lives

AE Hosman, HL Devlin BSc, BM Silva and CL Shovlin

Supplementary Data

Extracts from the SurveyMonkey[1] questionnaire at www.imperial.ac.uk/medicine/HHTsurvey2012

See reference [Silva et al, 2013] for further details

[1]SurveyMonkey.com, LLC, Palo Alto, California, USA (www.surveymonkey.com)

Content

Information and consent issues .... 3 -4

Personal Ascertainment of HHT Status.. 5-9

Family Ascertainment of HHT Status... 10

Relatives' data

- Brothers/sisters... 11-13
- Parents 14
- Grandparents 15
- Wider family (not counted as "cases") 16-17

Cancer Risk Factor Ascertainment 18-20

12/EM/0073: HHT and other medical conditions

The study is being performed by HHTIC London, the centre for people with hereditary haemorrhagic telangectasia (HHT) based at Hammersmith Hospital, which is part of Imperial College Healthcare NHS Trust, in London, England. The study has been reviewed by the NRES Committee East Midlands- Derby 1 Research Ethics Committee in England.

HHT is short for hereditary haemorrhagic telangiectasia (also known as Osler-Weber-Rendu syndrome, or Osler's disease). We have conducted many research studies to look into different medical problems that people with HHT experience, most recently for blood clots and during plane flights. By getting a better picture of medical problems that people with HHT do or do not have, we are able to provide the HHT community with better advice about their lifestyle and health, which may be reassurance, or possibly measures that can be taken to help improve health.

We invite you to help us with this research, by filling out the enclosed short questionnaire about general health issues. There are 5 sections, and it should take you about 20-30 minutes to fill in. This will allow us to see if people with HHT are more or less likely to have certain common medical conditions, than people without HHT. All questionnaires will be treated with the strictest of confidence. We will not disclose your personal results to your family doctor, or any third party.

It is up to you to decide whether to fill in the questionnaire. If you do fill it in and press the "DONE" button on the last page, we will accept your consent to use your answers in our research. We aim to publish our conclusions in a scientific journal. No study participant will be identified in any publication. Thank you very much for considering this invitation.

Dr Claire Shovlin,
Lead Clinician, HHTIC London

Comment box:

Note that to reduce bias, participants were provided with very general information in their information sheet, and the online survey introduction.

12/EM/0073: HHT and other medical conditions

1. ELECTRONIC CONSENT: Please select your choice below. Clicking on the "agree" button below indicates that:

• you have read the above information

• you voluntarily agree to participate

• you are at least 18 years of age

If you click on the "disagree" button this means you do not want to do the survey.

- ○ agree
- ○ disagree

Online, answer logic was applied so that clicking "disagree" directed participants automatically to the final page of the survey , so that no questions could be viewed or completed.

In the paper version, an ethically approved letter allowed us to contact individuals who had answered subsequent questions, but had not ticked "agree," to enable them to consent if that was their intention.

12/EM/0073: HHT and other medical conditions

SECTION 3: MEDICINES AND LIFESTYLE

In this section, we are looking briefly at factors that might affect the medical conditions we are interested in. Don't forget, that you can click here for the Participant Information Sheet, and that you can go backwards and forwards to check or correct most of your answers.

***110. First, can you please remind us, do you have or think you have HHT?**

- No, I do not have HHT — If ticked, participant was directed to non HHT question set and skipped section
- Yes, I do have HHT — If ticked, participant was directed to question 111
- I don't know — If ticked, participant was directed to non HHT question set and skipped section

Online, answer logic was applied as indicated.

In the paper version, participants were given text instructions on which question to proceed to.

12/EM/0073: HHT and other medical conditions

*3. Lots of people have nosebleeds or red spots on their skin. For people with HHT, these are in certain patterns. We want to know which of these statements about you are correct. Please tick all that apply to you.

- ☐ I have never had a nosebleed
- ☐ I have or had nosebleeds
- ☐ ....as a child
- ☐ .... after a knock or injury to the nose
- ☐ ....less than 5 times in my life
- ☐ ....about once a year
- ☐ ....at least once a month
- ☐ ....at least once a week
- ☐ ....at least once a day

If ticked, this was used as a “nosebleed” criterion

- ☐ I have noticed, or have been told by a doctor, that I have red spots on my tongue or in my mouth
- ☐ I have noticed, or have been told by a doctor, that I have red spots somewhere else ....
- ☐ ...... on my chest, back, or stomach
- ☐ ...... on my arms or legs
- ☐ ....on my face
- ☐ ....on my lips
- ☐ ....on the tips or pads of some of my fingers, on the opposite side to my nails

If ticked, this was used as “telangiectasia” criterion

12/EM/0073: HHT and other medical conditions

4. AVMs are abnormal blood vessels that affect people with HHT more commonly than people without HHT. AVM is short for "arteriovenous malformation". Which of these statements about you and AVMs are correct?

Please tick all that apply to you.

- ☐ I had never heard of AVMs before
- ☐ I have never been told I have an AVM
- ☐ I have or had AVMs, and these were ......
- ☐ ......AVMs in the lung (pulmonary AVMs)
- ☐ ......AVMs in the brain (cerebral AVMs)
- ☐ ......AVMs in the liver (hepatic AVMs)
- ☐ ......AVMs somewhere else in my body

If ticked, this was used as respective “AVM” criteria

Please say where any other AVMs were.

12/EM/0073: HHT and other medical conditions

7. This is a list of other possible specialist interventional treatments for HHT.

Please tick any of these treatments that you have had yourself.

- ☐ Nose bleed treatment by a hospital specialist
- ☐ Skin telangiectasia ("blood spot") treatment by skin doctors (dermatologists) or other hospital specialists
- ☐ GI/gut bleeding - laser treatment or surgery
- ☐ Lung (pulmonary) AVM - embolization or surgery
- ☐ Brain (cerebral) AVM - embolization, surgery, or radiotherapy
- ☐ Liver (hepatic) AVMs - specialist treatment
- ☐ I have not had any treatments like this.

If ticked, this was used as criterion

If relevant, please provide the names of any treatments- you have a maximum of 3 lines to use.

12/EM/0073: HHT and other medical conditions

***8. Have you had any of these specialist interventional/procedural treatments for HHT nosebleeds?**

Please tick any of these treatments that you have had yourself.

- ☐ Emergency packing by a hospital specialist
- ☐ Cauterisation/silver nitrate treatment
- ☐ Laser treatment
- ☐ Skin graft (Septal dermatoplasty)
- ☐ Nostril closure (Young's procedure)
- ☐ Ligation treatment of blood vessels to nose
- ☐ Embolisation treatment of blood vessels to the nose
- ☐ No, I have not had anything like this

If ticked, this was used as “nosebleed” criterion

Please tell us about any other treatments.

12/EM/0073: HHT and other medical conditions

*** 177. Are you from a family where you or other people have HHT?**

- ◯ No
- ◯ Yes, and we're sure, or fairly sure, that it's from my blood line (parents, grandparents etc)
- ◯ Yes, it's not in my blood line, but affected someone who came into my family through marriage or partnership
- ◯ Yes, but we're not sure where it came from- it seemed to start suddenly and we can't be sure if any of our older relatives had it

178. Which side of your family do you think HHT might have come from?

- ◯ My mother's
- ◯ My father's
- ◯ Not sure
- ◯ It doesn't, it comes from my partner's side of the family
- ◯ As I told you, none of my family, or partner's family, seem to have HHT

This was used to allocate HHT and non HHT arms to families

12/EM/0073: HHT and other medical conditions

184. Please tell us about any brothers and sisters that you have. We know that most of these lines will probably be left empty for you, but we want to make it as easy as possible to fill in for people who come from very big families.
(One of the options for all of these questions is "I'm not sure".)

	Brother or sister	Month of birth	Day of birth	Do/did they have HHT?	Have they had cancer?
Eldest brother or sister					
2nd brother or sister					
3rd brother or sister					
4th brother or sister					
5th brother or sister					
6th brother or sister					
7th brother or sister					
8th brother or sister					
9th brother or sister					
10th brother or sister					
11th brother or sister					
12th brother or sister					
Options for 20 provided					

DROPDOWN BOX OPTIONS:

- **No**
- **Yes**
- **Not sure**

if any of these are half brothers or sisters, please tell us which numbers these are

12/EM/0073: HHT and other medical conditions

186. If any of your brothers or sisters had cancer, roughly how old were they when they first had cancer? We do not need exact ages, just to the nearest 10-20 years, but one of the options is always "I'm not sure".

Here, 1st, 2nd and 3rd mean the brothers or sisters with cancer, not the eldest, 2nd eldest, 3rd eldest. Please leave blank if you have no brothers or sisters who had cancer.

	Their 1st cancer	Their 2nd cancer	Their 3rd cancer
1st brother or sister			
2nd brother or sister			
3rd brother or sister			

Please tell us the ages here if there were more than 3 brothers or sisters with cancer in either group

OPTIONS:
- Childhood (0-15)
- Late teens or 20s
- 30s
- 40s
- 50s
- 60s
- 70s
- 80s
- Later
- I'm not sure

These individuals were counted as HHTT or unknown as described elsewhere

12/EM/0073: HHT and other medical conditions

187. Still thinking about any brothers or sisters who had cancer, please use the drop down lists to tell us what type of cancer they had. One of the options for all of these questions is "I'm not sure".

Here, 1st, 2nd and 3rd mean the brothers or sisters with cancer, not the eldest, 2nd eldest, 3rd eldest. Please leave blank if you have no brothers or sisters who had cancer.

	Their 1st cancer	Their 2nd cancer	Their 3rd cancer
1st brother or sister			
2nd brother or sister			
3rd brother or sister			

Please tell us briefly if there were any others

DROPDOWN BOX OPTIONS:
- Brain
- Bladder
- Breast
- Cervical
- Colorectal
- Kidney
- Leukaemia
- Lymphoma/lymph glands
- Mesothelioma
- Mouth
- Myeloma
- Oesophagus
- Ovary
- Pancreas
- Prostate
- Skin (melanoma)
- Skin (not a melanoma)
- Stomach
- Uterus
- Other
- I'm not sure

These individuals were counted as HHT or unknown as described elsewhere

12/EM/0073: HHT and other medical conditions

188. For your parents, please use the drop down boxes to tell us what you can about them.

We realise you may not know exact details, so one of the options for all of these questions is "I'm not sure", and where we ask for ages, we do not need exact ages, just to the nearest 10-20 years.

	Month of birth	How old are they/were they?	Did/do they have HHT?			Did they have cancer?	If they had cancer, did this first appear in their...
Your mother							
Your father							

As on previous slide

189. If either of your parents had a cancer, please use the drop down boxes to tell us what type of cancer it was.

One of the options for all of these questions is "I'm not sure".

	Their 1st cancer	Their 2nd cancer	Their 3rd cancer
Your mother			
Your father			

As on previous slide

These individuals were counted as HHT, unknown, or non HHT cases (if the other parent was HHT affected) as described elsewhere

12/EM/0073: HHT and other medical conditions

190. For your 4 grandparents, please use the drop down boxes to tell us what you can about them.

We realise you may not know exact details, so one of the options for all of these questions is "I'm not sure", and where we ask for ages, we do not need exact ages, just to the nearest 10-20 years.

	How old are/were they?	Did/do they have HHT?			Did they have cancer?	If they had cancer, at what age did this first appear?
Your mother's mother						
Your mother's father						
Your father's mother						
Your father's father						

As on previous slide

191. If any of your grand-parents had a cancer, please use the drop down boxes to tell us what type of cancer it was

	Their 1st cancer	Their 2nd cancer	Their 3rd cancer
Your mother's mother			
Your mother's father			
Your father's mother			
Your father's father			

As on previous slide

These individuals were counted as HHT, unknown or non HHT cases (if the other grandparent was HHT affected) as described elsewhere

12/EM/0073: HHT and other medical conditions

209. Please tick the groups where someone in your wider family has had CANCER.

- ☐ I'm sorry, I don't know
- ☐ Uncles and aunts on my mother's side (blood relatives)
- ☐ Uncles and aunts on my father's side (blood relatives)
- ☐ Cousins on my mother's side
- ☐ Cousins on my father's side
- ☐ No one has had cancer that I know of

If you can, please tell us how many.

These data were used to capture wider "cancer-prone" families but the individuals were not counted as HHT or non HHT cases

12/EM/0073: HHT and other medical conditions

210. If you can, please tell us the number of your aunts, uncles and cousins who have had the following common cancers.

	None	1	2	3	4	5	more than 5
Brain	○	○	○	○	○	○	○
Breast	○	○	○	○	○	○	○
Cervical	○	○	○	○	○	○	○
Colorectal (lower gut)	○	○	○	○	○	○	○
Leukaemia or lymphoma (blood)	○	○	○	○	○	○	○
Lung	○	○	○	○	○	○	○
Prostate	○	○	○	○	○	○	○
Skin (maliognant melanoma)	○	○	○	○	○	○	○
Skin (not a melanoma)	○	○	○	○	○	○	○
Any other cancer	○	○	○	○	○	○	○

Please tell us about the other cancer if you can.

These data were used to capture wider "cancer-prone" families but the individuals were not counted as HHT or non HHT cases

12/EM/0073: HHT and other medical conditions

* 117. Have you used any hormone or antihormone treatments of any sort? This could be the oral contraceptive pill or hormone replacement therapy for women, anti-oestrogens such as tamoxifen, or hormonal treatment for men, such as that given for osteoporosis or prostate cancer.

- Yes
- No
- Not any more, but I used to in the past.

122. And if you have used female hormones, how long have you used them for?

- Less than 1 month
- Less than 6 months but more than 1 month
- Less than 1 year but more than 6 months
- 1-2 years
- 2-5 years
- More than 5 years

* 124. Have you used anti-androgens for any reason, such as for prostate cancer?

- Yes
- No
- Not any more but I used to

12/EM/0073: HHT and other medical conditions

*** 128. Have you used iron tablets?**

- ◯ Yes
- ◯ No
- ◯ Not any more, but I used to in the past.

*** 138. Have you used prednisolone, cortisone, hydrocortisone or other steroid tablets, injections, inhalers, sprays or creams? Please tick all that apply.**

- ☐ Yes, a steroid spray to my nose
- ☐ Yes, a steroid inhaler for my chest
- ☐ Yes, a steroid cream for my skin
- ☐ Yes, I have had steroid tablets or injections
- ☐ No, I have never had any steroid tablets, injections, inhalers or sprays

Please tell us why your doctor gave you steroids- we just want to know the name of the medical condition(s).

12/EM/0073: HHT and other medical conditions

143. Have you ever been sunburnt? Tick all that apply.

- ☐ Yes- my skin was red and sore, and peeled afterwards
- ☐ Yes- my skin was red, sore and blistered, and peeled afterwards
- ☐ Yes, I had all of this, and I developed a fever, or felt dizzy or sick
- ☐ No, I have never been sunburnt

*** 146. Do you smoke?**

- ◯ No
- ◯ Yes, I smoke
- ◯ I used to smoke but I have stopped
- ◯ No, but most of my life I've lived with a smoker

147. And if you are/were a smoker

	How many years did you smoke for?	What was your usual number per week?
Cigarettes	▾	▾
Cigars	▾	▾
Pipes	▾	▾
Other	▾	▾

How many years did you smoke for? options:
<1
1-5
5-10
10-15
15-20
20-25
25-30
30-35
35-40
40-45
45-50
50-55
55-60
>60

What was your usual number per week? options:
0
1
2
3
4
5
6
7
8
9
10
11-20
21-30
31-40
41-50
51-60
>60

12/EM/0073: HHT and other medical conditions

221. It is really important that we don't double count anybody. Could you please tell us where your parents and grandparents live, or last lived?

First please tell us the country where they live or last lived. Put "SAME" if it is the same as the person immediately above them in the list.

Your mother	
Your father	
Your mother's mother	
Your mother's father	
Your father's mother	
Your father's father	

222. Now please tell us the city or town where your parents and grandparents live or last lived. Put "SAME" if it is the same as the person immediately above them in the list.

Your mother	
Your father	
Your mother's mother	
Your mother's father	
Your father's mother	
Your father's father	

Two of the three questions to ensure relatives were not double counted. The third, the name of the oldest relative known to have HHT, would have been downloaded if required, but this was not necessay.
