# Supplementary material for: Specific cancer rates may differ in patients with hereditary haemorrhagic telangiectasia compared to controls
Source: Orphanet J Rare Dis. 2013 Dec 20;8:195. doi: 10.1186/1750-1172-8-195 (PMC3891994; doi:10.1186/1750-1172-8-195)
Supplement: Additional file 2 — Crude incidence of the 20 most common non skin cancers in survey respondents. [file 1750-1172-8-195-S2.docx]

|  | Controls | | | | HHT | | | | Mann Whitney |
| --- | --- | --- | --- | --- | --- | --- | --- | --- | --- |
|  | Total | Cancer cases | Cases per 100,000 | SD | Total | Cancer cases | per 100,000 | SD | p value |
| brain | 142 | 0 | 0 | 0 | 1007 | 2 | 199 | 4454 | 0.6 |
| bladder | 142 | 0 | 0 | 0 | 1007 | 3 | 298 | 5453 | 0.52 |
| breast | 142 | 2 | 1409 | 11826 | 1007 | 25 | 248 | 15570 | 0.43 |
| cervical | 142 | 2 | 1409 | 11826 | 1007 | 4 | 397 | 6293 | 0.18 |
| colorectal | 142 | 3 | 2113 | 14432 | 1007 | 9 | 894 | 9393 | 0.71 |
| kidney | 142 | 0 | 0 | 0 | 1007 | 1 | 99.3 | 3151 | 0.71 |
| leukemia | 142 | 0 | 0 | 0 | 1007 | 0 | 0 | 0 | .. |
| liver | 142 | 0 | 0 | 0 | 1007 | 0 | 0 | 0 | .. |
| lung | 142 | 1 | 704 | 8392 | 1007 | 5 | 497 | 7032 | 0.74 |
| lymphoma | 142 | 0 | 0 | 0 | 1007 | 6 | 596 | 7700 | 0.36 |
| malignant melanoma | 142 | 0 | 0 | 0 | 1007 | 1 | 99.3 | 3151 | 0.71 |
| mesothelioma | 142 | 0 | 0 | 0 | 1007 | 0 | 0 | 0 | .. |
| mouth | 142 | 0 | 0 | 0 | 1007 | 2 | 199 | 4454 | 0.6 |
| myeloma | 142 | 0 | 0 | 0 | 1007 | 0 | 0 | 0 | .. |
| oesophagus | 142 | 0 | 0 | 0 | 1007 | 0 | 0 | 0 | .. |
| ovary | 142 | 0 | 0 | 0 | 1007 | 4 | 397 | 6293 | 0.45 |
| pancreas | 142 | 0 | 0 | 0 | 1007 | 0 | 0 | 0 | .. |
| prostate | 142 | 0 | 0 | 0 | 1007 | 8 | 794 | 888 | 0.29 |
| stomach | 142 | 0 | 0 | 0 | 1007 | 0 | 0 | 0 | .. |
| uterus | 142 | 0 | 0 | 0 | 1007 | 2 | 199 | 4450 | 0.6 |
| COMBINED | 142 | 7 | 6400 | 25000 | 1007 | 75 | 7450 | 26300 | 0.65 |

eTable 1: Crude incidence of the 20 most common non skin cancers in respondents. SD: standard deviation
